# Association of the triglyceride-glucose index with the risk of sarcopenia: a meta-analysis with clinical implications

**DOI:** 10.1186/s12902-026-02315-w

**Published:** 2026-05-29

**Authors:** Jing Wei, Li Yuan, Linzhu Xiong, Lin Ruan, Rui Lin, Hongmei Jiang

**Affiliations:** Department of Geriatric Medicine, The Second People’s Hospital of Deyang, Deyang, China

**Keywords:** Triglyceride–glucose index, Sarcopenia, Risk, Meta-analysis

## Abstract

**Purpose:**

Sarcopenia is associated with adverse clinical outcomes in older adults, yet simple and widely available biomarkers for early risk identification remain limited. This study aimed to determine the relationship between the triglyceride-glucose (TyG) index and the risk of sarcopenia.

**Methods:**

PubMed, Web of Science and CNKI databases were searched up to October 27, 2025. Observational studies reporting the association between the TyG index and sarcopenia were included. Pooled odds ratios (ORs) with 95% confidence intervals (CIs) were calculated to evaluate the risk of sarcopenia associated with different TyG index levels, and subgroup analyses based on sex, age, comorbidity of diabetes mellitus and the TyG index grouping method were performed. All the statistical analyses were conducted with STATA 17.0 software.

**Results:**

Twelve studies involving 46,417 patients were included, with a sarcopenia prevalence of 12.2%. The pooled results demonstrated that a lower TyG index was significantly associated with an increased risk of sarcopenia (OR = 2.06, 95% CI: 1.65–2.58, *P* < 0.001). Subgroup analyses by sex (male: OR = 2.47, *P* = 0.012; female: OR = 2.00, *P* = 0.006), age (older: OR = 2.02, *P* < 0.001; younger: OR = 1.84, *P* = 0.117), comorbidity of diabetes mellitus (nondiabetic: OR = 2.79, *P* < 0.001) and grouping method by the TyG index (binary variable: OR = 2.14, *P* = 0.003; continuous variable: OR = 2.10, *P* < 0.001) revealed consistent results, indicating the significant association of TyG index with sarcopenia risk.

**Conclusion:**

The TyG index was significantly related to the prevalence of sarcopenia, and a lower TyG index indicated an increased risk of sarcopenia.

**Registration:**

International Platform of Registered Systematic Review and Metaanalysis Protocols No. INPLASY202640080, DOI 10.37766/inplasy2026.4.0080, https://inplasy.com/.

**Clinical trial number:**

Not applicable.

**Supplementary Information:**

The online version contains supplementary material available at 10.1186/s12902-026-02315-w.

## Introduction

Sarcopenia, characterized by the progressive loss of skeletal muscle mass and function, has become a major public health concern in aging societies. Recent epidemiological studies indicate that the prevalence of sarcopenia in community-dwelling older adults is approximately 10–20%, although it varies depending on the diagnostic criteria and study population [1]. Beyond being a marker of frailty, sarcopenia is independently associated with a wide range of adverse clinical outcomes, including falls, fractures, functional decline, postoperative complications, prolonged hospitalization, and increased mortality [2]. Established risk factors include advanced age, inadequate nutrition, physical inactivity, and chronic diseases such as diabetes and osteoporosis. Therefore, the identification of simple and accessible biomarkers for early detection and risk stratification of sarcopenia has become an important clinical priority [3]. Given the aging of populations and the increasing burden of chronic disease, identifying simple biomarkers or indices that help stratify the risk of sarcopenia in vulnerable groups is of considerable clinical importance.

The triglyceride–glucose (TyG) index, calculated from fasting triglyceride and plasma glucose levels, has emerged as a convenient and reliable surrogate marker of insulin resistance. Insulin resistance can impair muscle protein synthesis, reduce skeletal muscle glucose uptake, and promote chronic low-grade inflammation, all of which may contribute to muscle loss and functional decline, providing a biological rationale for investigating the relationship between the TyG index and sarcopenia. Compared with traditional methods such as the hyperinsulinemic–euglycemic clamp or the homeostasis model assessment of insulin resistance (HOMA-IR), the TyG index is inexpensive, reproducible, and easily obtained in routine clinical practice. Growing evidence has demonstrated that an elevated TyG index is associated with metabolic syndrome, type 2 diabetes, cardiovascular disease, and chronic kidney disease, highlighting its value as a systemic metabolic risk indicator [4, 5].

In recent years, an increasing number of observational studies have explored the relationship between the TyG index and sarcopenia; however, the findings have been inconsistent. Some cross-sectional analyses based on large population datasets such as the National Health and Nutrition Examination Survey (NHANES) reported that a higher TyG index was associated with an increased likelihood of sarcopenia, supporting the role of insulin resistance in muscle loss [6]. Conversely, other studies conducted in specific populations, including middle-aged and older women or individuals without diabetes, observed an inverse association, in which higher TyG levels appeared to be protective against sarcopenia, possibly reflecting differences in nutritional status, body composition, and metabolic reserve [7]. Furthermore, several investigations have suggested that composite indicators derived from the TyG index, such as TyG-BMI or TyG-waist circumference, may exhibit stronger associations with sarcopenia than the TyG index alone, indicating a complex interaction between metabolic dysfunction, adiposity, and skeletal muscle mass [8]. These heterogeneous findings, together with variations in study design, population characteristics, and diagnostic criteria for sarcopenia, make it difficult to draw definitive conclusions regarding the direction and magnitude of the association.

Despite the growing interest in the TyG index as a metabolic biomarker, no consensus has been reached regarding its relationship with sarcopenia, and the existing evidence has not been systematically synthesized. Therefore, this is the first meta-analysis to quantitatively evaluate the association between the TyG index and sarcopenia and to explore whether this relationship varies across different subgroups defined by sex, age, diabetes status, and TyG index classification methods.

## Methods

This study was performed in accordance with the PRISMA guidelines (2020) [9] **(**Supplementary file 1**)**. This study was registered in the International Platform of Registered Systematic Review and Meta-analysis Protocols: No. INPLASY202640080, DOI: 10.37766/inplasy2026.4.0080, https://inplasy.com/.

### Literature search

The PubMed, Web of Science and CNKI databases were searched up to October 27, 2025, with the following terms: triglyceride glucose index, TyG index and sarcopenia. The full search strategies for PubMed was provided in Supplementary file 2. Moreover, MeSH terms and free texts were used during the search process. References of included studies were also reviewed.

### Inclusion criteria

Studies that met the following criteria were included: (a) the TyG index was calculated according to the formula ln (triglyceride [mg/dL] × fasting glucose [mg/dL]/2) [10]; (b) the association between the TyG index and the risk of sarcopenia was explored; (c) odds ratios (ORs) with 95% confidence intervals (CIs) were reported; (d) studies were published in English or Chinese, as these were the primary languages accessible to the research team and covered the vast majority of publications in the searched databases; and (e) full texts were available and in cases where the full text of an article was unavailable, attempts were made to contact the corresponding authors; if this failed, the study was excluded from the analysis.

### Exclusion criteria

Studies that met the following criteria were excluded: (a) insufficient or severely duplicated (>50%) data; and (b) letters, editorials, reviews, case reports, animal studies or conference abstracts.

### Data extraction

The following data were collected from the included studies: first author, patient source, year, study design, sample size, number of sarcopenia cases, specific population characteristics, age, grouping method according to the TyG index, definition of sarcopenia, OR and 95% CI.

If a single publication reported results from more than one independent cohort, each cohort was treated as a separate dataset in the meta-analysis, provided that there was no overlap in participants.

### Quality evaluation

Methodological quality was evaluated by the Newcastle–Ottawa Scale (NOS), and studies with NOS scores ≥ 6 were defined as high-quality studies [11, 12].

Two independent reviewers (Jing Wei and Li Yuan) conducted the literature search, selection, data extraction and quality evaluation and any disagreements were resolved by team discussion.

### Statistical analysis

All analyses were conducted using STATA version 17.0 software. The primary outcome was the risk of sarcopenia associated with the TyG index. For studies reporting incidence or prevalence rates without directly reported odds ratios (ORs), we calculated ORs using standard formulas comparing the highest versus lowest TyG index categories or per unit increase, based on reported case numbers and total participants in each group. Heterogeneity between studies was assessed using the Q test and I² statistic. A random-effects model (DerSimonian and Laird method) was applied if significant heterogeneity was observed (I²>50% or *P* < 0.1); otherwise, a fixed-effects model (Mantel-Haenszel method) was used [13]. This approach ensures that between-study variability is appropriately accounted for in the presence of heterogeneity. Sensitivity analyses were conducted by sequentially omitting one study at a time to assess the robustness of the pooled results. Publication bias was evaluated using Begg’s funnel plot and Egger’s regression test, with *P* < 0.05 indicating potential bias. Subgroup analyses were performed based on sex, age, diabetes status, and TyG index classification to explore potential sources of heterogeneity [14, 15].

For studies that reported the number of sarcopenia cases and non-cases across TyG categories without directly providing ORs, crude ORs were calculated using a standard 2 × 2 contingency table as follows: OR=(a×d)/(b×c), where a and b represent the number of participants with and without sarcopenia in the lower TyG group, and c and d represent the corresponding numbers in the higher TyG group. When available, adjusted ORs reported from multivariable models were preferentially extracted.

## Results

### Literature selection process

**Fig. 1:**
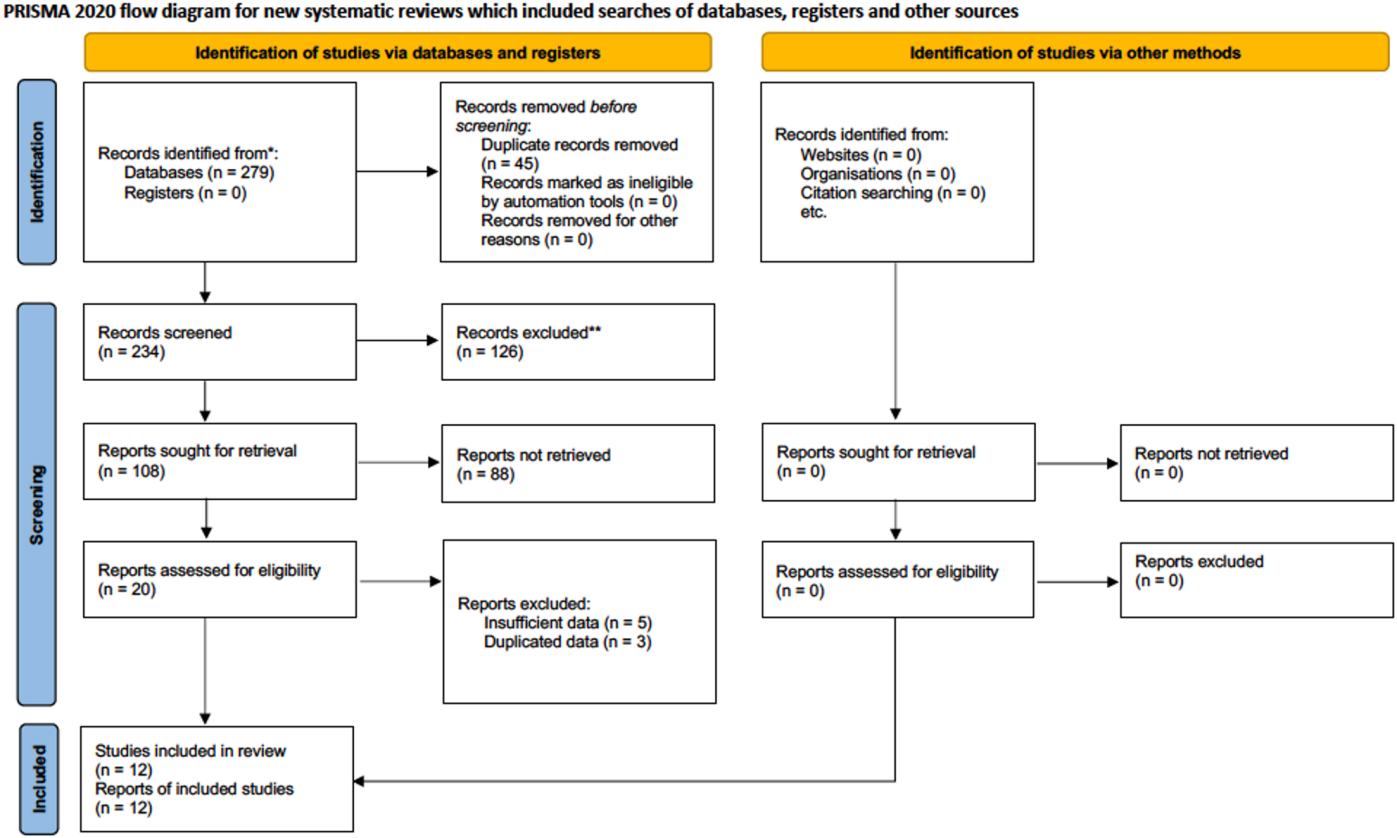
PRISMA flow diagram of this meta-analysis

Two hundred and seventy-nine publications were identified from the databases, and 45 duplicated publications were removed. As shown in Fig. 1, twelve publications comprising 13 independent datasets were eventually included after reviewing titles, abstracts and full texts [6, 7, 16–25].

### Basic characteristics

The basic characteristics of the included studies are shown in Table 1. A total of 46,417 participants from 13 independent datasets were included, and the prevalence rate of sarcopenia was 12.2% (5686/46417), with sample sizes ranging from 142 to 15,741. Four studies focused on nondiabetic patients, and five studies focused on elderly patients. In most studies, the TyG index was regarded as a continuous variable, and all the studies were high quality.

**Table 1:** Basic characteristics of included studies

Author	Year	Patient source	Study design	Sample size	Sarcopenia cases	Specific population	Age (year-old)	Grouping method by TyG index	Definition of sarcopenia	NOS
Ahn [16]	2020	Republic of Korea	Cross-sectional	15,741	1386	Non-diabetic	43.4 ± 0.2	TyG index <8.72 vs. TyG index >8.72	Male: ASM/BMI<0.789; female: ASM/BMI<0.512	8
Chen [17]	2022	China	Cross-sectional	142	40	Non-diabetic with MHD	54.05 ± 13.9	Continuous	Male: SMI<7.0 kg/m^2^; female: SMI<5.7 kg/m^2^	7
Chen [18]	2024	CHARLS	Cross-sectional	1819	217	None	≥ 45	Continuous	Male: SMI<6.797 kg/m^2^; female: SMI<4.950 kg/m^2^	8
Li [7]	2024	China	Cross-sectional	460	73	Non-diabetic women	≥ 50	Continuous	SMI<5.4 kg/m^2^ plus muscle strength <18 kg	8
Shi [19]	2024	China	Retrospective cohort	126	39	Non-diabetic with MHD	62.10 ± 13.47/ 55.89 ± 12.90	Continuous	Male: SMI<7.0 kg/m^2^; female: SMI<5.7 kg/m^2^	7
Yang J [6]	2024	NHANES (2011–2018)	Cross-sectional	4030	336	None	39.07 ± 11.77/42.96 ± 10.96	Continuous	Male: ASM/BMI<0.789; female: ASM/BMI<0.512	8
Yang X [20]	2024	NHANES (2011–2018)	Cross-sectional	981	90	CIAD	38.12 ± 12.94	Continuous/ TyG index <8.75 vs. TyG index >8.75	Male: ASM/BMI<0.789; female: ASM/BMI<0.512	8
Zhang [21]	2024	CHARLS	Cross-sectional	10,537	1602	None	≥ 45	Continuous	NR	8
Zhou [22]	2024	China	NR	2407	512	None	≥ 45	Continuous	Male: SMI<7.0 kg/m^2^; female: SMI<5.7 kg/m^2^	8
Zhang [23]	2025	NHANES (2011–2018)	Cross-sectional	827	116	CKD	46 (35–53)	Continuous	Male: ASM/BMI<0.789; female: ASM/BMI<0.512	8
Zhang [23]	2025	China	Cross-sectional	1038	178	CKD	46 (34–56)	Continuous	Male: ASM/BMI<0.789; female: ASM/BMI<0.512	8
Zhao L [24]	2025	China	NR	1148	276	OSA	66 (62–71)	Continuous	NR	8
Zhao R [25]	2025	NHNES (1999–2006, 2011–2018)	Cross-sectional	7161	821	None	41.77 ± 13.81	Continuous	Male: ASM/BMI<0.789; female: ASM/BMI<0.512	8

MHD: maintenance hemodialysis; TyG: triglyceride-glucose index; SMI: skeletal muscle mass index; CIAD: chronic inflammatory airway disease; CKD: chronic kidney disease; OSA: obstructive sleep apnea; NHANES: National Health and Nutrition Examination Survey; CHARLS: China Health and Retirement Longitudinal Study; NOS: Newcastle-Ottawa Scale, ASM: appendicular skeletal muscle mass; BMI: body muscle index

### Association of the TyG index with the risk of sarcopenia

**Fig. 2:**
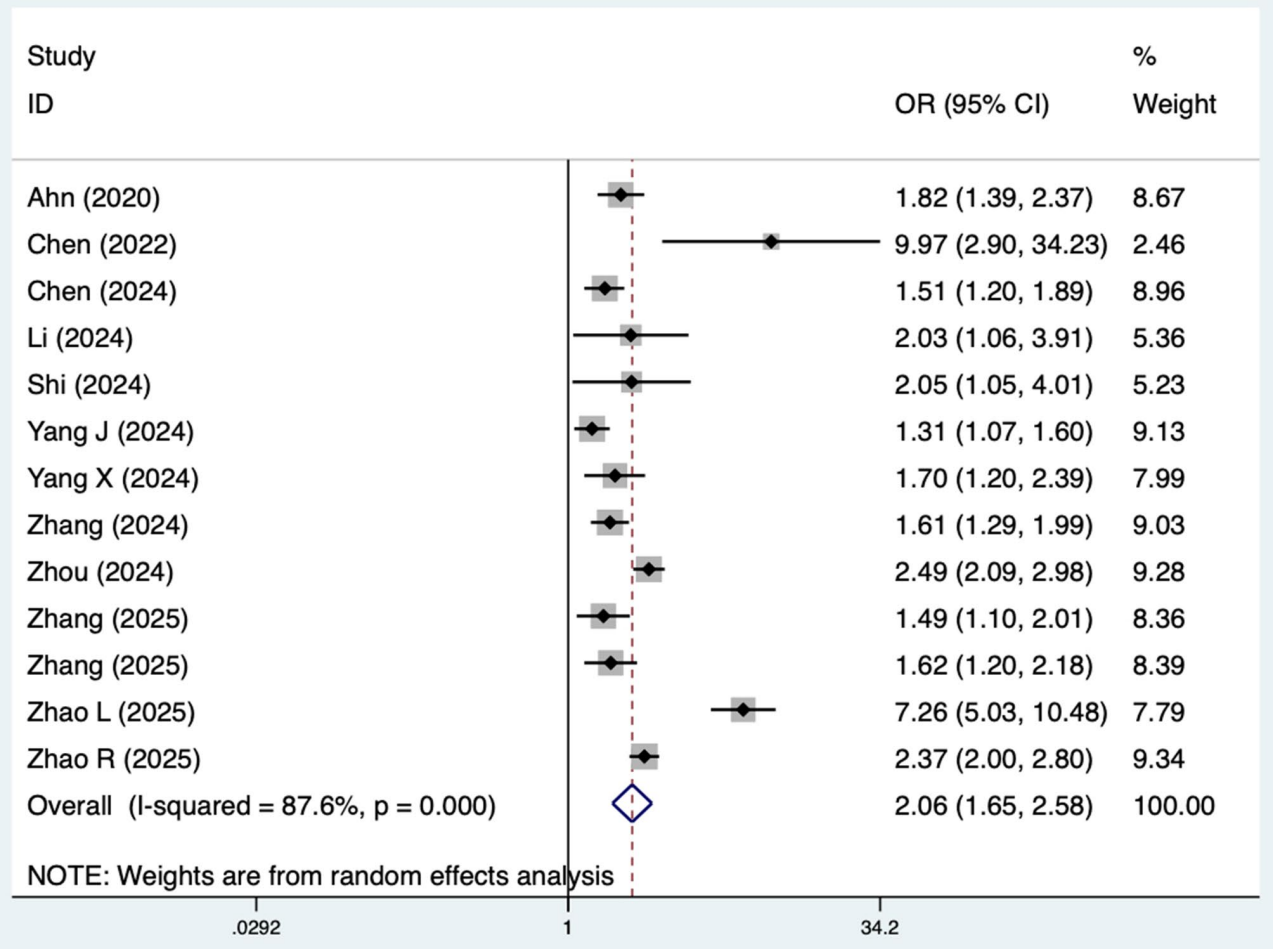
Association of triglyceride-glucose index with risk of sarcopenia

All included studies reported ORs directly; therefore, no recalculation from raw data was required. After combining the pooled results, it was demonstrated that the TyG index was significantly related to the risk of sarcopenia and that patients with a lower TyG index had a greater risk of sarcopenia (OR = 2.06, 95% CI: 1.65–2.58, *P* < 0.001; I^2^ = 87.6%, *P* < 0.001) (Fig. 2).

We subsequently conducted subgroup analyses based on the following parameters: sex (male: OR = 2.47, 95% CI: 1.22–4.99, *P* = 0.012; I^2^ = 92.9%, *P* < 0.001; female: OR = 2.00, 95% CI: 1.22–3.28, *P* = 0.006; I^2^ = 87.3%, *P* < 0.001) (Supplementary Fig. 1), age (older: OR = 2.02, 95% CI: 1.38–2.95, *P* < 0.001; I^2^ = 92.6%, *P* < 0.001; younger: OR = 1.84, 95% CI: 0.86–3.92, *P* = 0.117; I^2^ = 89.6%, *P* = 0.002) (Supplementary Fig. 2), comorbidity of diabetes mellitus (nondiabetic: OR = 2.79, 95% CI: 1.62–4.81, *P* < 0.001; I^2^ = 88.0%, *P* < 0.001) (Supplementary Fig. 3) and grouping method according to the TyG index (binary variable: OR = 2.14, 95% CI: 1.30–3.52, *P* = 0.003; I^2^ = 47.7%, *P* = 0.167; continuous variable: OR = 2.10, 95% CI: 1.64–2.68, *P* < 0.001; I^2^ = 88.6%, *P* < 0.001) (Supplementary Fig. 4), which further revealed the association between a lower TyG index and an increased risk of sarcopenia. Although the relationship between the TyG index and sarcopenia risk among younger patients was not statistically significant, the trend was quite obvious. However, the subgroup analyses for younger participants and for studies using binary TyG classification included only two studies each; therefore, these findings should be interpreted as exploratory and preliminary (Table 2).

**Table 2:** Results of meta-analysis

Items	Number of studies	Odds ratio	95% confidence interval	*P* value	I^2^ (%)	*P* value
Overall	12	2.06	1.65–2.58	< 0.001	87.6	< 0.001
Sex						
Male	4	2.47	1.22–4.99	0.012	92.9	< 0.001
Female	5	2.00	1.22–3.28	0.006	87.3	< 0.001
Age						
Older	7	2.02	1.38–2.95	< 0.001	92.6	< 0.001
Younger	2	1.84	0.86–3.92	0.117	89.6	0.002
Diabetes mellitus						
Non-diabetic	6	2.79	1.62–4.81	< 0.001	88.0	< 0.001
Grouping method by TyG index						
Binary variable	2	2.14	1.30–3.52	0.003	47.7	0.167
Continuous variable	11	2.10	1.64–2.68	< 0.001	88.6	< 0.001

TyG: triglyceride-glucose index

### Sensitivity analysis

**Fig.3:**
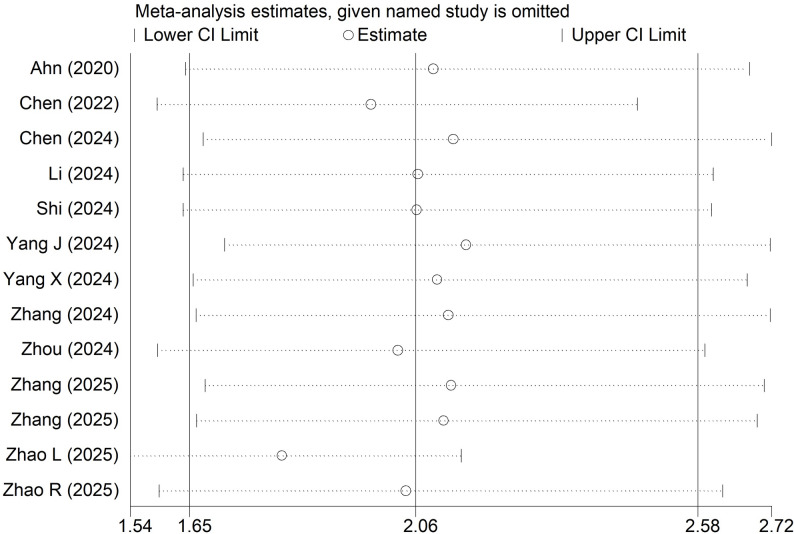
Sensitivity analysis about the association of triglyceride-glucose index with risk of sarcopenia

The sensitivity analysis indicated that our pooled results were stable and reliable. None of the included studies affected the overall results (Fig. 3).

### Publication bias

**Fig. 4:**
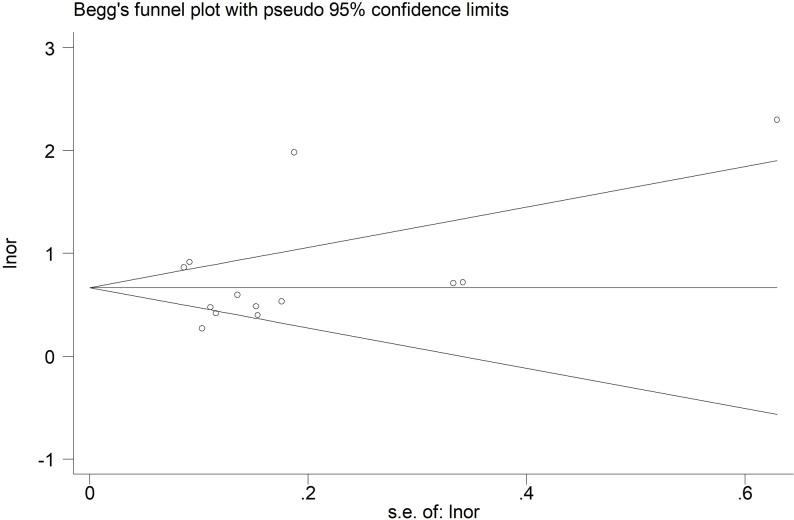
Begg’s funnel plot about the association of triglyceride-glucose index with risk of sarcopenia

Begg’s funnel plot (Fig. 4) and Egger’s test (*P* = 0.493) did not indicate statistically significant publication bias; however, given the limited number of included studies (~ 12), these tests are underpowered, and potential publication bias cannot be definitively ruled out.

## Discussion

In this meta‑analysis of twelve observational studies involving 46,417 participants, we found that a lower TyG index was significantly associated with an increased risk of sarcopenia (pooled OR = 2.06, 95% CI: 1.65–2.58, *P* < 0.001), suggesting that the TyG index may serve as a useful clinical marker for sarcopenia risk stratification. The pooled OR of 2.06 suggests that individuals with lower TyG index have approximately a two-fold higher likelihood of sarcopenia compared with those with higher TyG levels. From a clinical perspective, such a magnitude of association is meaningful, particularly given the simplicity and routine availability of TyG-related laboratory parameters in daily practice. This finding highlights that the TyG index is significantly associated with sarcopenia and may serve as a useful metabolic marker in clinical settings for identifying individuals with lower muscle mass or function. However, given that most included studies are cross-sectional, this association does not establish temporality or causality [18, 26].

Although the TyG index is generally recognized as a marker of insulin resistance and metabolic dysfunction, our finding that a lower TyG index is associated with increased sarcopenia risk can be explained by population-specific factors. In older adults or chronically ill populations, a low TyG index may reflect poor nutritional status, systemic catabolism, or a “consumptive” phenotype characterized by low circulating triglyceride and glucose levels. Such metabolic states can directly contribute to muscle wasting and sarcopenia, providing a biologically plausible explanation for the observed inverse association. This nuanced interpretation highlights that the clinical meaning of the TyG index may differ depending on the health and nutritional status of the population studied [25, 27, 28].

Importantly, the apparent “directional reversal” observed in this meta‑analysis should not be interpreted as contradictory to the insulin‑resistance hypothesis, but rather as reflecting population‑dependent metabolic contexts. In metabolically unhealthy but nutritionally preserved populations, higher TyG levels may indicate insulin resistance and contribute to muscle dysfunction [29, 30]. In contrast, in older or chronically ill populations with lower energy reserves (e.g., CKD, hemodialysis, OSA), lower TyG levels may reflect reduced caloric intake, systemic inflammation, or catabolic states, which are themselves closely linked to muscle wasting [31]. Due to the limited number of studies within each specific clinical subgroup, formal meta‑regression or stratified analyses by detailed disease categories were not statistically feasible. However, the substantial heterogeneity observed across studies likely reflects these clinically distinct metabolic phenotypes. Future studies with larger numbers of homogeneous cohorts are needed to clarify whether the TyG–sarcopenia association differs fundamentally across specific clinical contexts [17, 31].

Our main finding of an inverse association between low TyG index and sarcopenia risk complements prior individual studies that have reported both positive and inverse relationships between TyG and muscle outcomes. For example, large cross‑sectional NHANES analyses have demonstrated a positive association between higher TyG index and sarcopenia prevalence in U.S. adults, with per‑unit TyG increases associated with higher sarcopenia risk (OR = 1.31) after multivariable adjustment, suggesting insulin resistance may promote muscle loss in general populations [6]. Conversely, some studies in specific subgroups such as nondiabetic populations or elderly cohorts reported inverse associations, potentially reflecting the influence of nutritional status and energy reserves on metabolic markers and muscle mass — a phenomenon which could explain discrepancies between studies conducted in different populations and health contexts [6, 25].

Subgroup analyses stratified by sex revealed that the inverse association between the TyG index and sarcopenia risk was present in both males and females, although the effect size appeared somewhat larger in males than females. Biological sex differences in body composition, hormonal milieu, and insulin sensitivity may partly underlie this pattern, as men tend to have greater proportions of lean mass and different responses to metabolic insults than women [32]. Furthermore, previous work suggests that sex hormones and inflammatory mediators modulate muscle metabolism and insulin signaling differently in males and females, which may influence the TyG–sarcopenia relationship.

Regarding age, we found consistent associations in older adults but not statistically significant results among younger groups, possibly due to lower sarcopenia prevalence and smaller sample sizes in the latter subgroup, leading to reduced power to detect associations. Aging is accompanied by insulin resistance, chronic low‑grade inflammation, and anabolic resistance, which accelerate muscle catabolism and may amplify the connection between metabolic dysfunction and muscle loss in older persons. These biological processes have been highlighted in epidemiological studies linking insulin resistance and sarcopenia pathogenesis in aging cohorts [32, 33].

A notable finding of this meta-analysis is the substantial heterogeneity observed across studies (I² > 85% in most analyses). Although a random-effects model was applied to account for between-study variability, the high heterogeneity suggests that the pooled effect size should be interpreted cautiously. Several factors may have contributed to this variability. First, differences in diagnostic criteria for sarcopenia (e.g., EWGSOP, AWGS, and other definitions) involve distinct thresholds for muscle mass, strength, and physical performance, which may influence prevalence estimates and effect sizes. Second, variations in TyG index categorization (continuous vs. binary variables) and cut-off values may affect comparability across studies. Third, covariate adjustment strategies differed substantially, with some studies reporting multivariable-adjusted ORs and others providing crude estimates. In addition, the included studies comprised both cross-sectional and cohort designs, and were conducted in heterogeneous populations (community-based vs. hospital-based settings; differing age structures; varying comorbidity profiles such as CKD, OSA, or hemodialysis populations). Ethnic and regional differences may also contribute to metabolic and body composition variability. Collectively, these methodological and clinical differences likely explain a significant proportion of the observed heterogeneity.

This study has several limitations. First, significant heterogeneity was observed across included studies, which may arise from variations in study design, population characteristics, assessment methods, and definitions of sarcopenia and TyG classification. Second, the included studies comprised both cross-sectional and cohort designs, and the reported odds ratios were derived from either fully adjusted models or crude estimates depending on the original study. Although combining different study designs and adjustment models may limit causal inference and complicate interpretation, sensitivity analyses and consistent subgroup results indicate that the main association between lower TyG index and sarcopenia risk is robust. Although sensitivity analyses demonstrated that no single study unduly influenced the pooled estimate, the substantial heterogeneity warrants cautious interpretation of the overall effect size. Third, some subgroup analyses included fewer than three studies, limiting the robustness of estimates in these analyses. Fourth, TyG index categorizations and sarcopenia diagnostic criteria varied across the included studies, which may introduce heterogeneity and affect conceptual comparability. We addressed this by standardizing effect estimates (e.g., calculating ORs for highest vs. lowest TyG categories or per unit change) and conducting subgroup analyses based on TyG classification methods. Sensitivity analyses confirmed that the main association remained robust, suggesting that pooling across heterogeneous definitions did not materially compromise the validity of our findings. Fifth, although the included studies enrolled both diabetic and non-diabetic participants, none of the studies specifically reported results for diabetic subpopulations. Consequently, our subgroup analyses could only focus on non-diabetic participants, and the association between the TyG index and sarcopenia in diabetic patients could not be assessed. This limits the generalizability of our findings to diabetic populations. Moreover, some subgroup analyses (e.g., younger participants and binary TyG classification) were based on only two studies, which limits the robustness and reliability of these pooled estimates. Finally, the majority of studies were cross‑sectional in design, which precludes causal inference and highlights the need for prospective cohort data to clarify temporal relationships. Our search was restricted to studies published in English and Chinese, which may introduce language bias and limit the generalizability of our findings.

Although a formal GRADE assessment was not performed, the overall certainty of evidence for the primary outcome should be interpreted as moderate to low. Most included studies were observational in design (predominantly cross-sectional), which inherently limits causal inference. In addition, substantial heterogeneity (high I² values), variability in diagnostic criteria, and differences in adjustment strategies may further reduce confidence in the pooled estimates. Therefore, while the observed association appears consistent in direction, the strength of evidence should be interpreted cautiously, and well-designed prospective studies are needed to confirm these findings.

Considering emerging evidence, future research should focus on prospective cohort studies and clinical trials to determine whether changes in the TyG index over time predict incident sarcopenia and whether interventions targeting metabolic pathways (e.g., improving insulin sensitivity or nutritional supplementation) can modify sarcopenia risk. Additionally, exploring TyG‑related indices such as TyG‑BMI, TyG‑WC or TyG‑WHtR that combine anthropometric and metabolic factors may improve predictive accuracy for sarcopenia beyond the TyG index alone, as suggested by recent multicohort studies [21]. Integration of TyG monitoring into routine geriatric assessment frameworks could support early preventive strategies tailored to individual metabolic profiles.

## Conclusion

In summary, this meta‑analysis demonstrates a robust association between lower TyG index values and increased sarcopenia risk. These findings underscore the potential of the TyG index as a pragmatic biomarker for identifying individuals at risk for sarcopenia in a variety of clinical settings and populations. Incorporating the TyG index alongside clinical, functional, and nutritional assessments may help identify individuals with lower muscle mass or function and inform further evaluation, although causal inference cannot be made from the current observational evidence. However, prospective studies and standardized definitions are needed to validate optimal TyG thresholds and clarify mechanistic pathways underlying the TyG–sarcopenia relationship.

## Supplementary Information

Below is the link to the electronic supplementary material.

Supplementary Material 1: Supplementary Fig. 1. Subgroup analysis by the sex about the association of triglyceride-glucose index with risk of sarcopenia.

Supplementary Material 2: Supplementary Fig. 2. Subgroup analysis by the age about the association of triglyceride-glucose index with risk of sarcopenia.

Supplementary Material 3: Supplementary Fig. 3. Subgroup analysis by the comorbidity of diabetes mellitus about the association of triglyceride-glucose index with risk of sarcopenia.

Supplementary Material 4: Supplementary Fig. 4. Subgroup analysis based on the grouping method by triglyceride-glucose index about the association of triglyceride-glucose index with risk of sarcopenia.

Supplementary Material 5

Supplementary Material 6

## Data Availability

All data used in this meta-analysis were presented in the manuscript.

## Abbreviations

TyG: Triglyceride-glucose
OR: Odds ratio
CI: Confidence interval
NHANES: National Health and Nutrition Examination Survey
NOS: Newcastle–Ottawa Scale
