# Supplementary material for: Association of the triglyceride-glucose index with the risk of sarcopenia: a meta-analysis with clinical implications
Source: BMC Endocr Disord. 2026 May 29;26:220. doi: 10.1186/s12902-026-02315-w (PMC13436063; doi:10.1186/s12902-026-02315-w)
Supplement: Supplementary file 2 [file 12902_2026_2315_MOESM6_ESM.docx]

**PubMed:**
